# Construction and effectiveness of a pharmacist-involved diabetes management model between tertiary hospitals and community under the hierarchical medical system

**DOI:** 10.3389/fcdhc.2025.1658713

**Published:** 2025-09-19

**Authors:** Nan Gao, Linyan Lan, Zizhen Jia, Huaying Li, Xiangxiang Xie, Han Xie, Cheng Ji

**Affiliations:** ^1^ Department of Pharmacy, China Pharmaceutical University, Nanjing Drum Tower Hospital, Nanjing, China; ^2^ Department of Pharmacy, Taicang Loujiang New City Hospital, Suzhou, China; ^3^ Department of Pharmacy, Inner Mongolia Medical University, Hohhot, China; ^4^ Department of Pharmacy, Xishanqiao Community Health Service Center, Nanjing, China; ^5^ Department of Pharmacy, Nanjing Drum Tower Hospital, Affiliated Hospital of Medical School, Nanjing University, Nanjing, China

**Keywords:** type 2 diabetes, chronic disease management, pharmacy services, community health centers, hierarchical medical system

## Abstract

**Objective:**

This study constructed a tertiary hospital-community health service center diabetes linkage management model with the participation of clinical pharmacists, assessed the changes in clinical indicators and medication treatment of patients with type 2 diabetes before and after the implementation of the model, and evaluated the model, with a view to providing a model reference in the participation of clinical pharmacists in the management of type 2 diabetes and other chronic diseases. Given the current situation that diabetes management at the community level in China is still unsatisfactory, with an HbA1c control rate of less than 10% compared to about 50% in tertiary hospitals, there is an urgent need to explore innovative, pharmacist-involved models to bridge this gap.

**Methods:**

Using the principle of randomization, patients who met the enrollment criteria were divided into the experimental group and the control group. A total of 210 patients were enrolled from three community health service centers in Nanjing in collaboration with Drum Tower Hospital, and were followed up for 12 months. Clinical indicators and medication adherence were used as evaluation endpoints to compare the differences in management effects between the two groups. This study was registered with the Chinese Clinical Trial Registry (ChiCTR2300074444).

**Results:**

Under the diabetes linkage management model, patients in the intervention group showed improvement in blood glucose, glycated hemoglobin and other indicators compared with the control group; the medication adherence score of patients in the intervention group was significantly higher than that of the control group.

**Conclusion:**

The clinical efficacy and medication level of diabetic patients were significantly improved after management by this management model, which provides a reference for clinical pharmacists to carry out pharmacy services in the context of hierarchical diagnosis and treatment. This model may contribute to narrowing the quality gap between tertiary hospitals and community health service centers in diabetes care.

## 1 Introduction

Diabetes, a global epidemic, is experiencing a rapid increase in prevalence, with projections suggesting a rise to 700 million by 2045 (1–3). Epidemiological surveys conducted in China from 2015 to 2017 indicate a prevalence rate of 11.2% among adults, with newly diagnosed diabetes and prediabetes at 6.8% and 35.2%, respectively (4). Particularly alarming is the control of diabetes at the community level, where awareness, treatment, and glycemic control rates stand at 38.6%, 35.6%, and 33.0% respectively—figures that urgently need improvement (5). It is critical to note that with an HbA1c control target of<7%, the community compliance rate is less than 10%, a stark contrast to the 50% observed in tertiary hospitals.

Pharmacists, as important members of chronic disease management teams, have been shown in international studies to improve patients’ glycemic control, self-management abilities, and medication adherence through interventions such as medication therapy management and diabetes self-management programs (6–12). However, in China, community-level pharmaceutical services started late, are still underdeveloped, and are often replaced by physicians or nurses, leading to an unmet need for pharmacist-led interventions in primary care settings (13).

In collaboration with the Yuhuatai District Health Commission, Drum Tower Hospital, and three community health service centers governed by the Yuhuatai District Health Commission in Nanjing, our research group has established a medical consortium. Based on this platform, we have developed a hospital–community linkage model, a diabetes management framework that actively integrates clinical pharmacists into the continuum of care between tertiary hospitals and community health centers. This model draws on both international experiences (such as the Chronic Care Model and Transitional Care Model) (14, 15) and national health policies encouraging hierarchical diagnosis and treatment (16), and it aims to fill the gap of inadequate pharmaceutical care in community diabetes management (13). The rationale for pharmacist involvement in diabetes management can also be explained through behavioral theories. The Health Belief Model (17) highlights the role of perceived benefits, barriers, and cues to action in influencing adherence behaviors. Pharmacist-led counseling and continuous follow-up provide clear cues to action and reduce barriers by addressing medication concerns. Similarly, Social Cognitive Theory emphasizes self-efficacy, which is essential for chronic disease self-management (18). By providing education, skills training, and reinforcement, pharmacists enhance patients’ confidence to adhere to medications and engage in lifestyle modification. Therefore, the hospital–community linkage model is not only aligned with system-level frameworks such as the Chronic Care Model and Transitional Care Model (19, 20), but also grounded in behavioral theories that explain how pharmacist interventions improve clinical outcomes (21).

Using a randomized controlled trial, this study investigates the efficacy of this model in managing Type 2 Diabetes Mellitus (T2DM) patients, aiming to enhance the management outcomes and ensure the effectiveness and safety of pharmacological treatments. This delineates a reference framework for involving pharmacists in the coordination between tertiary hospitals and community health services under a hierarchical medical structure, particularly for chronic ailments like T2DM.

## 2 Materials and methods

To gauge the efficacy of the integrated management system in diabetes treatment, researchers implemented a randomized controlled study. T2DM patients were enrolled from key community health stations within Yuhuatai District, Jiangbei New District, and Gulou District of Nanjing. Participants were randomly assigned to either a “Clinical Pharmacist-Involved Treatment Group” or a “Traditional Doctor Treatment Group,” with both groups undergoing a 12-month follow-up. The efficacy of the model was assessed based on changes in clinical indicators, medication regimens, and diabetes self-management capabilities before and after the intervention. The study received ethical approval from the Ethics Committee of Drum Tower Hospital (Ethical Approval Number: 2020-233-02) and was publicly registered with the Chinese Clinical Trial Registry (Registration Number: ChiCTR2300074444). Adherence to the Helsinki Declaration’s ethical principles was paramount in this trial, ensuring full protection of subjects’ safety, legal rights, and personal information throughout the course of the study.

### 2.1 Inclusion and exclusion criteria

Inclusion Criteria: (1) Patients aged ≥18 years who meet the T2DM diagnostic criteria as per the “National Primary Diabetes Prevention and Management Guidelines” (2021 Edition); (2) Patients who are treated at community health service centers and cooperate with management interventions and subsequent follow-ups; (3) Individuals capable of self-managing their lifestyle; (4) Individuals who are able to engage in physical activities; (5) Individuals with the ability to hear, speak, read, write, and communicate effectively; (6) Patients informed about the study objectives, willing to participate, and having endorsed an informed agreement form.

Exclusion Criteria: (1) Subjects with significant hepatic or renal dysfunction; (2) Patients who are unconscious or unable to communicate effectively; (3) Patients with significant organ failure or severe coronary heart disease, cerebrovascular disease, or heart failure, or those who have had a myocardial infarction in the past 12 months; (4) Patients with a severe history of neurological or psychiatric disorders; (5) Patients with severe infections; (6) Patients with active disseminated intravascular coagulation (DIC); (7) Patients with malignant tumors; (8) Patients with alcoholism and drug addiction; (9) Pregnant or lactating women; (10) Subjects lacking a signed declaration of consent after being informed about the study.

Sample Size Calculation

The sample size was calculated using the following formula:


$$n=\frac{{\left(\mu_{\alpha }+\mu_{\beta }\right)}^{2}\cdot 2p1-p}{{\left(p_{1}-p_{2}\right)}^{2}}$$

Glycemic control rate among community-dwelling patients with diabetes was one of the primary outcomes of this study. According to the literature (22), using HbA1c< 7% as the criterion for glycemic control, the rate among Chinese community patients with diabetes is less than 10%, whereas the overall control rate among adult patients receiving treatment is approximately 40%. It was assumed that the glycemic control rate in the intervention group would reach 40% after 12 months of the intervention. The Type I error probability (α) was set at 0.05 (μ_α_ = 1.96), and the Type II error probability (β) at 0.20 (power = 1 – β = 0.80, μ_β_ = 1.282). With an allocation ratio of 1:1 between the intervention and control groups, p_1_ (control) = 0.10, p_2_ (intervention) = 0.40, and the pooled proportion (p) = 0.25, the minimum required sample size was calculated to be 128 patients (64 per group). To account for potential loss to follow-up and incomplete data, we planned to recruit approximately 200 patients. In practice, 210 patients were enrolled across three community hospitals, which exceeded the calculated requirement.

**Figure 1:**
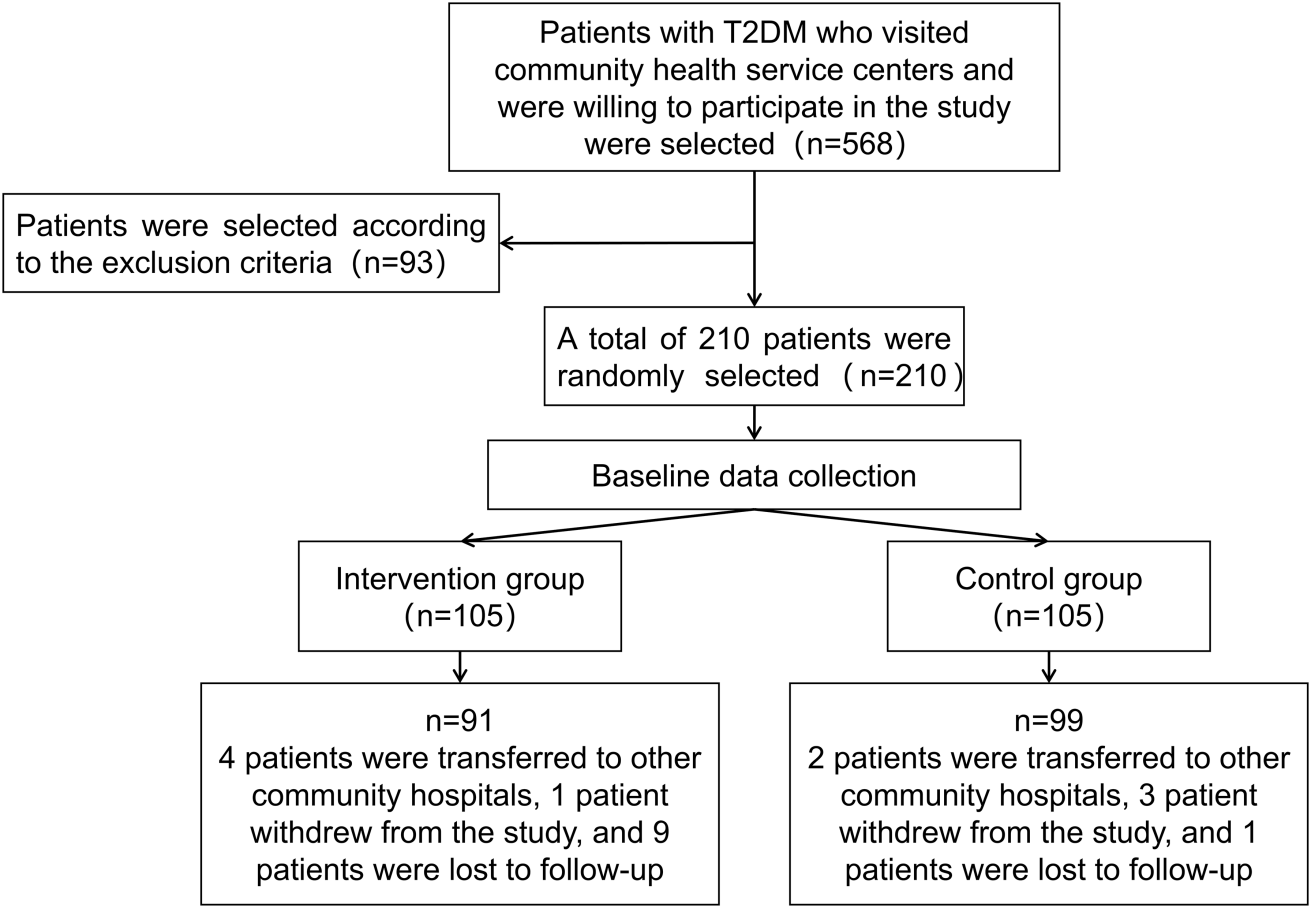
Randomized controlled trial patient enrollment flowchart.

Patients in the trial group received management under the clinical pharmacist-involved model, while the control group receives traditional doctor management. Both groups of patients are followed up according to the predefined clinical protocols, as illustrated in **Figure 1**.

### 2.2 Randomization and allocation

Randomization was conducted using a computer-generated random number table, and eligible patients were subsequently allocated in a 1:1 ratio to the intervention and control groups. Outcome assessors and data analysts were blinded to group allocation in order to reduce the risk of assessment bias.

### 2.3 Specific management measures

Patients in the control group received routine doctor-led care and diabetes education through scheduled seminars. These covered topics such as the clinical manifestations of diabetes, treatment methods, prevention of complications, blood glucose monitoring, diabetes care, dietary management in diabetes, and exercise management. Patients were contacted for follow-up visits at 3, 6, 9, and 12-month intervals via phone calls.

On top of routine physician management, the intervention group received pharmacist-led collaborative care: (1) Baseline Assessment–One-on-one evaluation of current medications, glycemic control, lifestyle, and adherence. (2) Pharmaceutical Care–Identification and classification of drug-related problems (DRPs) using the *Pharmaceutical Care Network Europe* classification system (PCNE-DRP V8.03), with individualized recommendations reviewed by physicians. (3) Follow-up–Monthly pharmacist follow-ups via clinic or telephone, with additional visits if needed. (4) Adherence Support–Regular assessment using the *8-item Morisky Medication Adherence Scale* (MMAS-8); targeted counseling was provided to improve adherence. (5) Self-management education included standardized training in blood glucose monitoring, medication use, diet, and exercise. Self-care was assessed using the Summary of Diabetes Self-Care Activities (SDSCA) questionnaire (see **Supplementary Table S1**). Diabetes knowledge was evaluated with the Diabetes Knowledge Test (DKT), for which the Chinese version translated and adapted by Ai-Ling Chen was used (see **Supplementary Table S2**).

**Figure 2:**
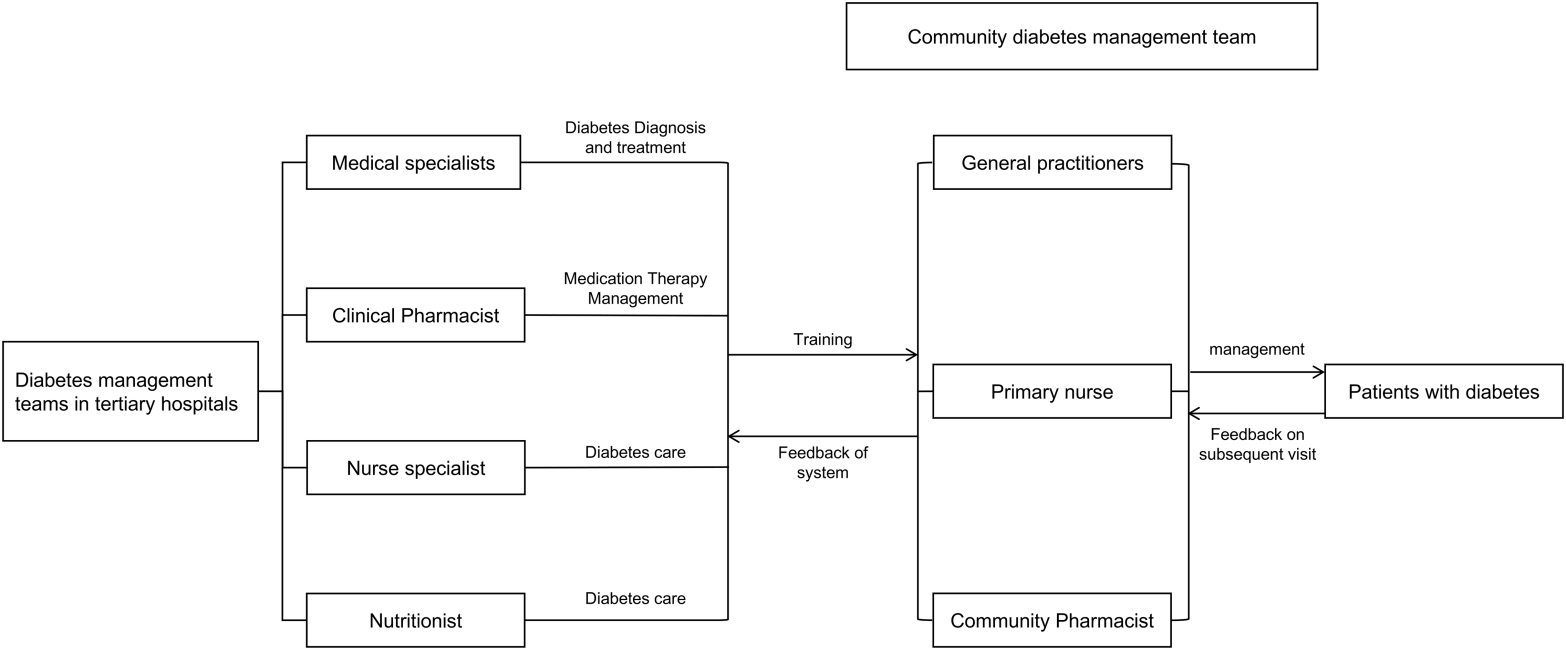
Diabetes management mechanism linking tertiary and community hospitals.

To ensure standardized and consistent implementation of the intervention across the three participating centers, a unified training and credentialing system for community pharmacists was established under the coordination of the Pharmaceutical Affairs Division of the Yuhuatai District Health Commission. Expert faculty from the Department of Pharmacy at Nanjing Drum Tower Hospital provided centralized instruction and practical supervision; on this basis, pharmacists from the tertiary hospital conducted multiple rounds of specialized, targeted training for community pharmacists (1–3 sessions per module). Training was delivered primarily through online and in-person lectures, with face-to-face teaching and hands-on practicums for practice-oriented skills, to ensure close alignment between training content and job requirements. Through this mechanism, the intervention was implemented across centers in a consistent, standardized, and reproducible manner (see **Supplementary Table S3** for an overview of the specialized training for community pharmacists). The integrated tertiary–community hospital diabetes management mechanism is illustrated in **Figure 2**.

For both groups, initial and follow-up visit data were meticulously recorded and updated in health records for subsequent data analysis.

### 2.4 Observation indicators

Hemoglobin A1c (HbA1c) was designated as the primary endpoint of this trial. Other outcomes were regarded as secondary or exploratory, and consequently, adjustments for multiple comparisons were not undertaken.

#### 2.4.1 Clinical indicators

①Biochemical indicators: Fasting Plasma Glucose (FPG), Hemoglobin A1c (HbA1c), triglycerides, total cholesterol, high-density lipoprotein cholesterol, and low-density lipoprotein cholesterol; ②Blood Pressure; ③Body Mass Index (BMI); ④Analysis of changes from baseline to endpoint in glycemic control, blood pressure control, and lipid control rates in both the experimental and control groups.

#### 2.4.2 Patient medication management

Diabetes patients require ongoing adjustments to their treatment regimens based on glycemic control and overall health, with timely medication changes enhancing treatment adherence and reducing adverse drug interactions. In this study, clinical pharmacists utilized the PCNE-DRP V8.03 classification system to categorize and analyze drug-related problems (DRPs), propose intervention plans, report to the general practitioners in charge of treatment, and document the physicians’ acceptance of pharmacist interventions. The validated 8-item Morisky Medication Adherence Scale (MMAS-8) was employed to evaluate patients’ compliance with prescribed medications (23), as shown in **Table 1**. An analysis was conducted on changes in the types and quantities of medications, medication adherence, the incidence of DRPs in the experimental group, and the acceptance of medication interventions by doctors and patients before and after management in both groups. This analysis aims to confirm the effectiveness of pharmacist interventions in the medication management of diabetes patients.

**Table 1:** The 8-item Morisky medication adherence scale (MMAS-8) questionnaire.

Items	Option (score)	
(1) Do you sometimes forget to take your glucose-lowering medications?	Yes (0)	No (1)
(2) Over the past two weeks, were there any days when you did not take your glucose-lowering medications?	Yes (0)	No (1)
(3) Have you ever cut back or stopped administration without telling your doctor because you felt worse when you took it?	Yes (0)	No (1)
(4) When you travel or leave home, do you sometimes forget to take your glucose-lowering medications?	Yes (0)	No (1)
(5) Do you ever intentionally skip taking your medication?	Yes (0)	No (1)
(6) When you feel like your diabetes is under control, do you sometimes stop taking your medicine?	Yes (0)	No (1)
(7) Use your glucose-lowering medications every day is a real inconvenience for some people. Do you ever feel hassled about sticking to your diabetes treatment plan?	Yes (0)	No (1)
(8) How often do you have difculty remembering to take your glucose-lowering medications?	Never (1) Almost never (0.75) Sometimes (0.5) Quite often (0.25) Always (0)	

The Morisky Medication Adherence Scale (MMAS-8) was used under formal license from Donald E. Morisky, ScD, MMAS Research LLC. Documentation of licensing approval is provided in the **Supplementary Materials**.

### 2.5 Data analysis

All analyses were conducted on the per-protocol population. Missing data were handled using a complete-case approach, whereby participants with missing values were excluded from the corresponding analyses, rather than imputed (e.g., by last observation carried forward or multiple imputation). After the collection and screening of clinical data and questionnaires were completed, the data were double-entered into the EpiData V.3.1 database and then analyzed statistically using SPSS version 23.0. The normality of continuous variables was assessed using the Kolmogorov–Smirnov (KS) test. Normally distributed continuous variables were expressed as mean ± SD. Inter-group comparisons utilized independent samples *t*-tests, while intra-group analyses employed paired samples *t*-tests. Median values characterized continuous variables without normal distribution. The Mann-Whitney U test was applied for between-group comparisons, while the Wilcoxon signed-rank test was used for within-group analyses. Percentages represented categorical variables, which were subjected to χ2 test analysis. Statistical significance was defined as *p*<0.05.

## 3 Results

### 3.1 Descriptive statistics of patients

Patient enrollment began in May 2021 for two months. The follow-up duration was 12 months, with all patients completing their follow-up by June 2022. A total of 210 eligible patients who agreed to participate were divided into the experimental group (*n*=105) and the control group (*n*=105). Twenty patients dropped out during the follow-up, resulting in 91 patients in the experimental group and 99 in the control group being included in the statistical analysis. The average ages of the experimental group and the control group were 66.01 ± 7.38 years and 65.96 ± 8.81 years, respectively, consistent with the demographics of elderly patients primarily seen in community hospitals (24). According to the SDSCA scale guidelines, No notable disparities were observed between the two groups of patients in adherence to regular dietary habits and regular exercise habits without management. Baseline demographic data of the patients in both groups, including age, gender, duration of illness, family history of diabetes, smoking, alcohol consumption, diet, exercise, and complications (hypertension, hyperlipidemia), showed no significant differences (*P*>0.05). Blood glucose levels showed no statistically meaningful variations between the two groups, blood pressure, lipid levels, and BMI indicators (*P*>0.05). Detailed data can be found in **Table 2**.

**Table 2:** Baseline characteristics of patients.

Variable	Intervention group (n=91)	Control group (n=99)	*P value*
Basic information			
Age, mean (SD)	66.01 (7.38)	65.96 (8.81)	0.661
Gender			0.678
Male	35 (38.19)	41 (41.41)	
Female	56 (60.81)	58 (58.58)	
Course of disease, n (%)			0.523
≤5 years	59 (64.80)	54 (54.54)	
6–10 years	15 (16.40)	23 (23.23)	
10–20 years	15 (16.40)	19 (19.19)	
>20 years	2 (2.19)	3 (3.03)	
Marital status, n (%)			0.892
Married	82 (90.1)	91 (91.9)	
Others	9 (9.8)	8 (8.0)	
Family history of diabetes (previous generation), n (%)			0.685
Both parents	3 (3.29)	5 (5.05)	
Father or mother	23 (25.27)	31 (31.31)	
Neither	59 (64.83)	63 (63.63)	
Smoke, n (%)			0.924
Yes	27 (29.67)	30 (30.30)	
No	64 (70.32)	69 (69.69)	
Drink, n (%)			0.225
Yes	18 (19.78)	27 (27.27)	
No	73 (80.21)	72 (72.72)	
Regular diet, n (%)	43 (47.25)	46 (46.46)	0.913
Regular exercise, n (%)	53 (58.24)	63 (63.63)	0.446
Hypertension, n (%)			0.993
Yes	41 (45.05)	44 (44.44	
No	50 (54.94)	55 (55.55)	
Hyperlipidemia, n (%)			0.418
Yes	43 (47.25)	41 (41.41)	
No	48 (54.94)	58 (58.58)	
Medications, n (%)			0.115
Yes	42 (53.80)	57 (57.57)	
No	49 (46.20)	42 (42.42)	
Clinical indicators, mean/median ± standard deviation			
BMI, kg/m^2^	25.12 ± 3.08	24.64 ± 3.00	0.336
FPG, mmol/L	8.42 (7.15, 10.54)	8.36 (6.98, 9.94)	0.259
HbA_1c_, %	7.70 (6.80, 10.05)	8.00 (7.10, 9.35)	0.763
DBP, mmHg	79.73 (73.0, 87.2)	78.49 (73.0, 86.0)	0.373
SBP, mmHg	133.50 (127.0, 145.0)	135.50 (125, 150)	0.452
TC, mmol/L	5.04 (4.21, 5.35)	4.65 (3.47, 5.64)	0.249
TG, mmol/L	1.76 (1.76, 2.43)	1.57 (0.97, 2.32)	0.191
HDL-C, mmol/L	1.12 (0.97, 1.26)	1.09 (0.95, 1.29)	0.062
LDL-C, mmol/L	2.96 (2.19, 3.37)	2.62 (1.98, 3.23)	0.733

### 3.2 Clinical indicator results

#### 3.2.1 Blood glucose levels

**Figure 3:**
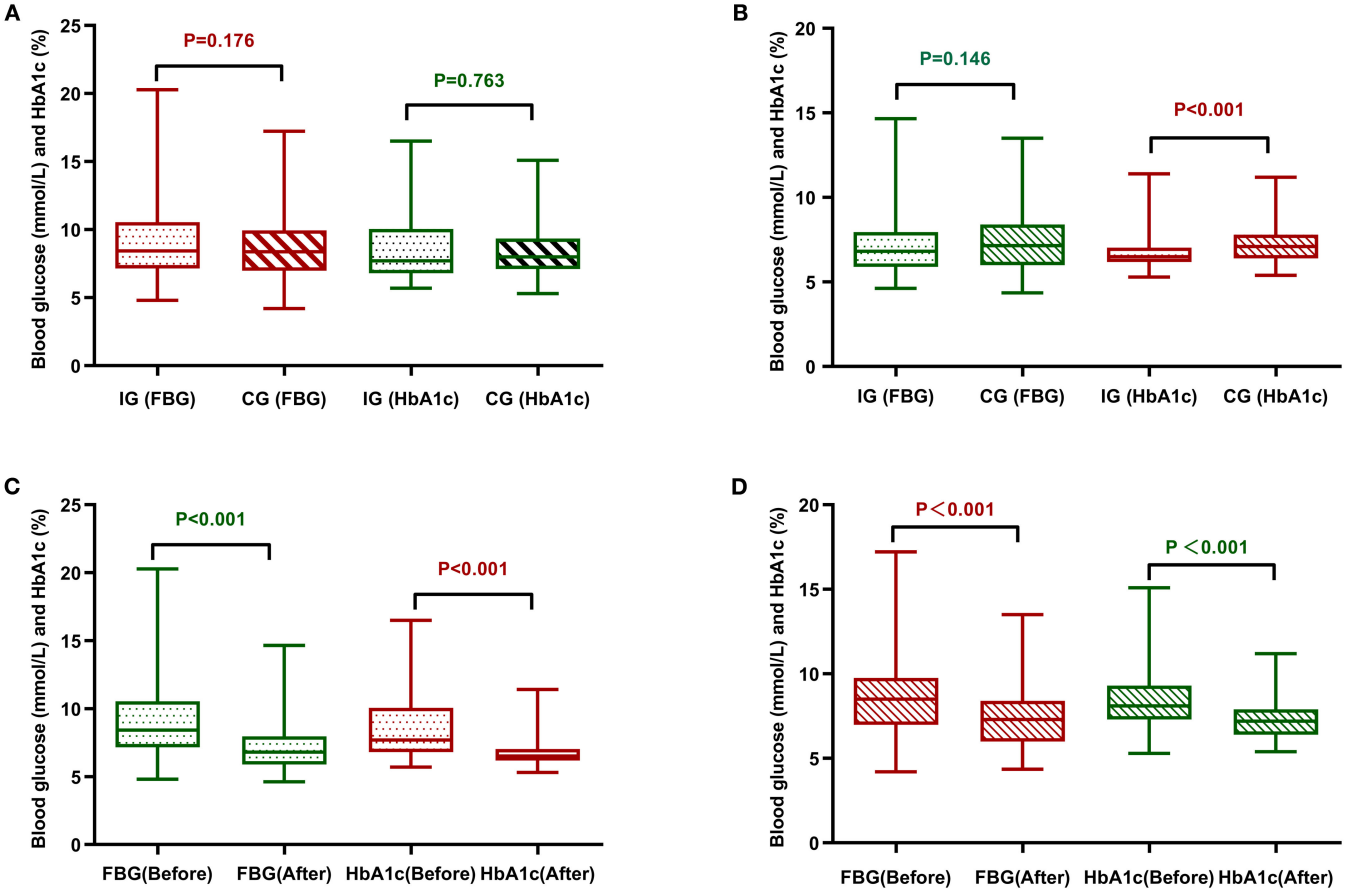
Comparative analysis of FPG and HbA1c between the two groups of patients. **(A)** Comparison of the two groups of patients before management. **(B)** Comparison of the two groups of patients after management. **(C)** Comparison before and after management of the intervention group. **(D)** Comparison of control group before and after management. CG, control group; IG, intervention group.

The main observational indicators were comparisons of changes in FPG (Fasting Plasma Glucose) and HbA1c levels between the two groups, as shown in **Table 3** and **Figure 3** Post-management data revealed a marked decrease in FPG and HbA1c concentrations for both cohorts, with statistical significance (*P*<0.001). Post-intervention comparisons of FPG levels between the experimental group and the control group showed that the decrease in FPG was slightly greater in the experimental group, but this difference was not statistically significant (*P*=0.146). Similarly, intergroup comparisons of HbA1c levels showed significant differences in the reductions before and after the intervention (*P*<0.001), indicating that during the same experimental period, the decrease in HbA1c was more pronounced in the experimental group than in the control group.

**Table 3:** Comparative analysis of glucose, blood pressure and lipids in two patient groups.

Variable	Clusters	Baseline	Follow-up	*Z*	*P*	*r (95%CI)*
FPG (mmol/L)	Intervention group	8.42 (7.15, 10.54)	6.81 (5.89, 7.95)	-6.76	<0.001	−0.709 (−0.800, −0.589)
	Control group	8.36 (6.98, 9.94)	7.15 (6.00, 8.40)	-4.94	<0.001	−0.497 (−0.633, −0.333)
	Z	-1.353	-1.45			
	*p*	0.176	0.146			
	*r (95%CI)*	−0.098 (−0.236, 0.044)	−0.105 (−0.244, 0.037)			
HbA_1c_ (%)	Intervention group	7.70 (6.80, 10.05)	6.50 (6.17, 7.02)	-6.32	<0.001	−0.662 (−0.764, −0.528)
	Control group	8.00 (7.10, 9.35)	7.10 (6.40, 7.80)	-6.48	<0.001	−0.651 (−0.752, −0.519)
	Z	-0.302	-3.51			
	*p*	0.763	<0.001			
	*r (95%CI)*	−0.022 (−0.163, 0.121)	−0.255 (−0.383, −0.117)			
SBP (mmHg)	Intervention group	133.50 (127.00, 145.00)	123.00 (125.00, 150.00)	-6.346	<0.001	−0.666 (−0.767, −0.531)
	Control group	135.50 (125.00, 150.00)	136.80 (128.20, 145.00)	-1.359	0.324	−0.137 (−0.326, 0.062)
	Z	-0.752	-5.653			
	*p*	0.452	<0.001			
	*r (95%CI)*	−0.055 (−0.195, 0.088)	−0.410 (−0.521, −0.284)			
DBP (mmHg)	Intervention group	79.73 (73.00, 87.20)	76.50 (70.70, 84.00)	-2.156	0.031	−0.226 (−0.414, −0.020)
	Control group	78.49 (73.00, 86.00)	76.40 (70.00, 82.00)	1.684	0.096	0.169 (−0.029, 0.355)
	Z	0.894	-0.393			
	*p*	0.373	0.695			
	*r (95%CI)*	0.065 (−0.078, 0.205)	−0.029 (−0.170, 0.113)			
TC (mmol/L)	Intervention group	5.04 (4.21, 5.35)	4.28 (3.70, 5.04)	-3.206	0.001	−0.336 (−0.507, −0.138)
	Control group	4.65 (3.47, 5.64)	4.38 (3.28, 5.25)	-2.390	0.017	−0.240 (−0.417, −0.045)
	Z	-1.153	-0.092			
	*p*	0.249	0.927			
	*r (95%CI)*	−0.084 (−0.223, 0.059)	−0.007 (−0.149, 0.135)			
TG (mmol/L)	Intervention group	1.76 (1.76, 2.43)	1.08 (0.78, 1.83)	-2.739	0.006	−0.287 (−0.466, −0.086)
	Control group	1.57 (0.97, 2.32)	1.30 (0.89, 2.28)	-2.587	0.010	−0.260 (−0.434, −0.066)
	Z	-1.308	-1.518			
	*p*	0.191	0.129			
	*r (95%CI)*	−0.095 (−0.233, 0.048)	−0.110 (−0.249, 0.032)			
HDL-C (mmol/L)	Intervention group	1.12 (0.97, 1.26)	1.27 (1.08, 1.49)	-2.625	0.009	−0.275 (−0.456, −0.072)
	Control group	1.09 (0.95, 1.29)	1.21 (1.00, 1.42)	-2.192	0.028	−0.220 (−0.400, −0.024)
	Z	-0.341	-1.131			
	*p*	0.733	0.258			
	*r (95%CI)*	−0.025 (−0.166, 0.117)	−0.082 (−0.221, 0.061)			
LDL-C (mmol/L)	Intervention group	2.96 (2.19, 3.37)	2.45 (1.89, 2.89)	3.220	0.002	0.338 (0.141, 0.509)
	Control group	2.62 (1.98, 3.23)	2.38 (1.76, 3.36)	1.399	0.166	0.141 (−0.058, 0.329)
	Z	1.876	0.637			
	*p*	0.062	0.525			
	*r (95%CI)*	0.136 (−0.006, 0.273)	0.046 (−0.098, 0.188)			

Using HbA1c ≤ 6.5% as the standard level, the changes in the proportion of patients achieving target blood glucose levels before and after management in the two groups were analyzed, as shown in **Table 4**. Using the corrected chi-square test, the proportion of individuals in the experimental arm who achieved optimal glycemic targets differed significantly between pre- and post-intervention periods (*P<* 0.001). Using the McNemar exact test, a noteworthy change was observed in the control arm’s results following the intervention, with the difference achieving statistical significance (*P<* 0.001). However, the number of patients in the control group whose blood glucose levels remained below the target both before and after management was as high as 70.7% (70 cases), and the proportion of patients whose blood glucose did not improve to the target level was substantially higher compared to the experimental group, which was at 5.4%, indicating that the management plan of the experimental group was significantly effective in improving the rate of patients reaching target blood glucose levels.

**Table 4:** Comparative analysis of blood-glucose in two groups of patients.

Clusters			Follow-up		χ^2^	*P*
			Standard	Non-standard		
Intervention group	Baseline	standard	9	3	22.400	<0.001
		non-standard	32	47		
Control group	Baseline	standard	10	1	13.474	<0.001
		non-standard	18	70		

#### 3.2.2 Blood pressure levels

**Figure 4:**
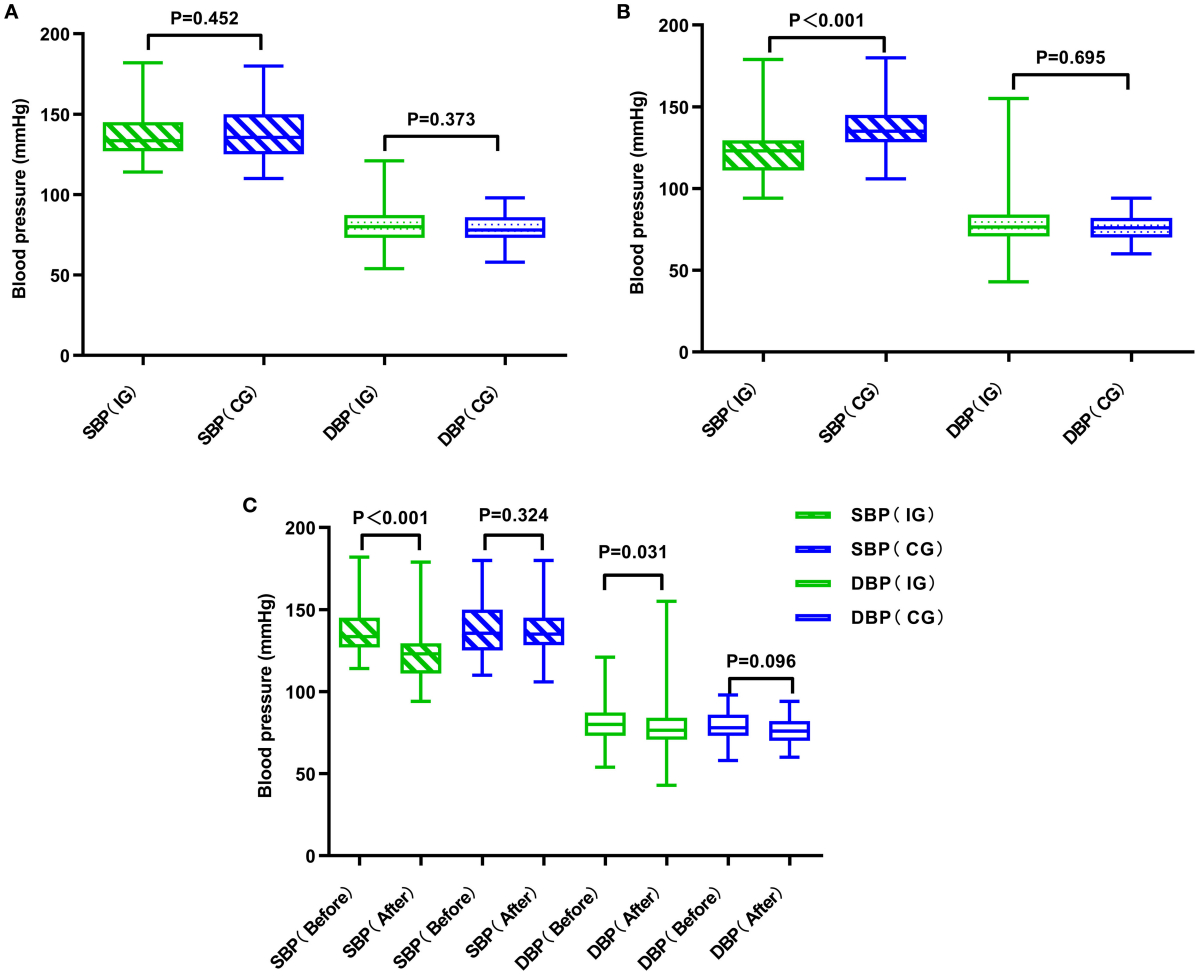
Comparative analysis of blood pressure in two groups of patients. **(A)** Comparison of the two groups of patients before management. **(B)** Comparison of the two groups of patients after management. **(C)** Comparison of the two groups of patients before and after management. CG, control group; IG, intervention group.

**Table 3** and **Figure 4** illustrate the blood pressure alterations observed in both groups following the management intervention. In the experimental group, both systolic blood pressure (SBP) and diastolic blood pressure (DBP) showed significant decreases (*P*< 0.001 vs *P* = 0.031). In contrast, the decreases in SBP and DBP in the control group were not statistically significant (*P* = 0.324 vs *P* = 0.096). A comparison of post-intervention SBP and DBP values between the two groups revealed that the decrease in SBP was significantly more pronounced in the experimental group (*P*< 0.001), no substantial divergence was noted in the magnitude of DBP reduction when comparing the two study populations (*P* = 0.695).

#### 3.2.3 Lipid levels

Overall, both management models improved lipid levels in patients, except for the change in LDL-C (Low-Density Lipoprotein Cholesterol) in the control group, which was not significantly different. The experimental group did not demonstrate a significant advantage in lipid improvement over the control group. Specific results are detailed in **Table 3**.

#### 3.2.4 Body mass index

Compared to baseline, the experimental group’s BMI decreased by 0.71 kg/m² (*P*=0.001), whereas the control group’s BMI decreased by 0.33 kg/m² (*P*=0.518). Both groups of patients were overweight before management, and although the results show a statistically significant reduction in BMI in the experimental group post-intervention, it still did not reach below-normal levels. Results are shown in **Table 5**.

**Table 5:** Comparative analysis of body mass index between the two groups of patients.

Variable	Clusters	Baseline	Follow-up	*t*	*P*	*r (95%CI)*
BMI (kg/m^2^)	Intervention group	25.12 ± 3.08	24.41 ± 6.29	-3.228	0.001	−0.322 (−0.496, −0.123)
	Control group	24.64 ± 3.00	24.31 ± 2.69	-0.647	0.518	-0.065 (−0.259, 0.134)
	t	-0.963	-0.254			
	*p*	0.336	0.799			
	*r (95%CI)*	-0.070 (−0.210, 0.073)	-0.019 (−0.161, 0.123)			

#### 3.2.5 “3B” achievement rate

The “3B” achievement rate, which refers to the simultaneous achievement of target levels for blood glucose, blood pressure, and lipids, was analyzed. Results, as evidenced in **Table 6**, the baseline achievement rates exhibited no statistically significant disparity across the studied groups (*P*=0.457). A substantial disparity was observed in the achievement rates from baseline to follow-up in the experimental group (*P*=0.001); however, the variation in achievement rates pre- and post-management in the control group did not reach significance (*P*=0.082). Post-intervention, the experimental group saw a significant escalation in the number of individuals meeting the 3B standards relative to the control group (*P*=0.007).

**Table 6:** Achievement of the “3Bs” before and after management of patients in both groups.

Clusters	Intervention group	Control group	c^2^	*P*	*OR (95%CI)*
Baseline	20	16	0.554	0.457	1.46 (0.70–3.03)
follow-up	41	26	7.335	0.007	2.30 (1.25–4.23)
c^2^	10.874	3.022			
*P*	0.001	0.082			

### 3.3 Medication treatment

#### 3.3.1 Medication adherence scoring

To assess the standardization of patient medication usage, the Morisky Medication Adherence Scale was administered to both study groups. Initially, both groups displayed low adherence levels (*P*=0.842). After 12 months of intervention, the score in the experimental group increased from 5.75 to 7.00 (*P*<0.001), while in the control group it rose from 5.75 to 6.00 (*P*<0.001). The experimental group demonstrated a markedly superior improvement in scores from baseline to post-intervention, compared to the control group, with the difference reaching statistical significance (*P*<0.001).The scores are displayed in **Table 7**.

**Table 7:** Medication adherence scores of patients in both groups.

Variable	Clusters	Baseline	Follow-up	t	*P*	*r (95%CI)*
Morisky	Intervention group	5.75 ± 1.30	7.00 ± 1.32	-6.828	<0.001	-0.584 (−0.705, −0.430)
	Control group	5.75 ± 1.16	6.00 ± 1.13	-5.171	<0.001	-0.463 (−0.605, −0.291)
	t	-0.199	-6.273			
	*P*	0.842	<0.001			
	*r (95%CI)*	-0.014 (−0.156, 0.128)	-0.415 (−0.528, −0.289)			

#### 3.3.2 Drug-related problems

**Figure 5:**
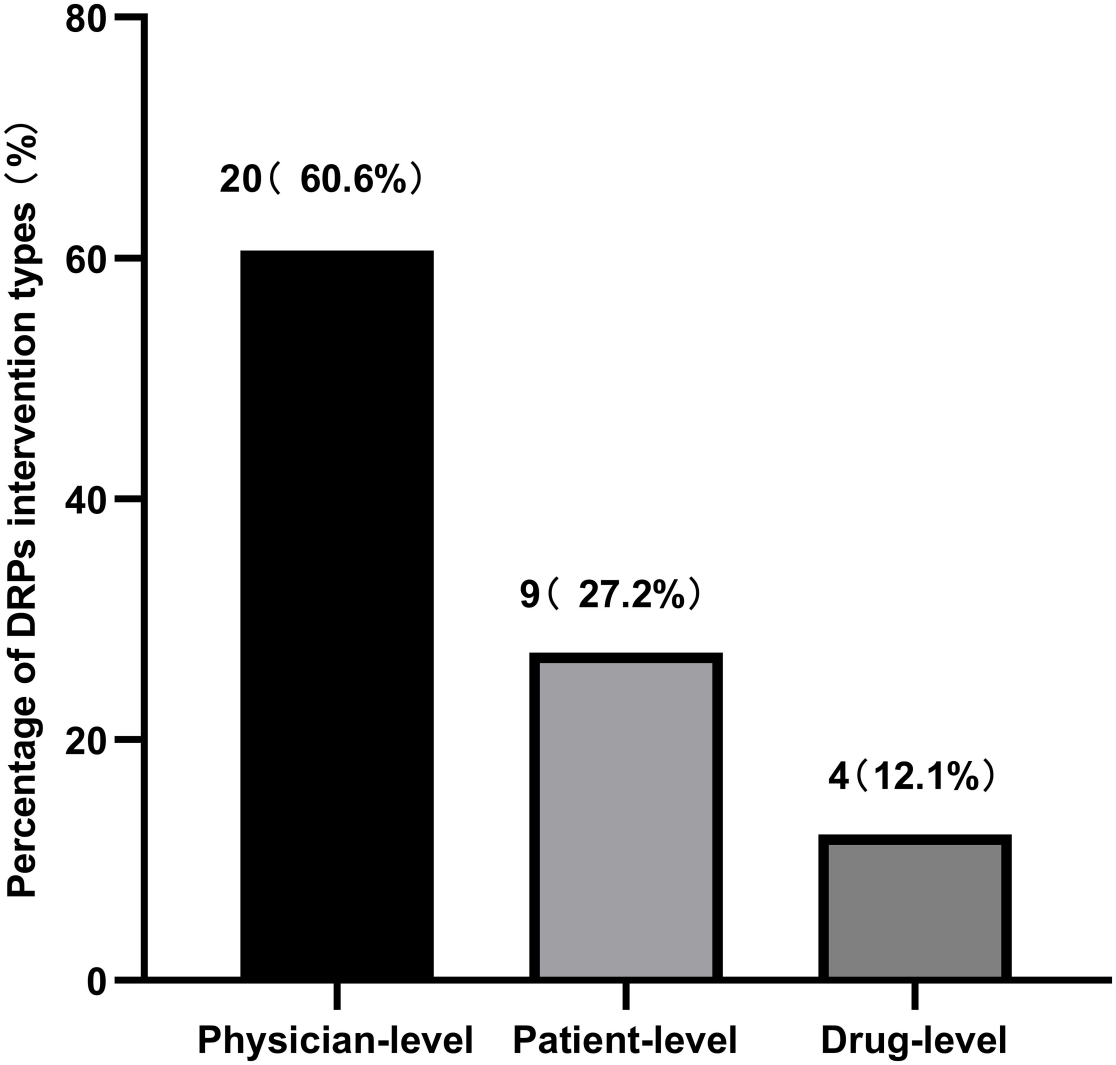
DRPs types of intervention.

During the treatment and follow-up periods, the incidence of DRPs in the experimental group was 37.3%. **Table 8** documents the occurrence rates of various DRPs, and **Table 9** lists the causes of these DRPs. The types of interventions for DRPs are shown in **Figure 5**.

**Table 8:** Type and percentage of DRPs.

Type of issue	Percentage, n (%)
Therapeutic efficacy	18 (54.5)
Ineffective medication	2
Poor treatment results	11
Presence of untreated symptoms or indications	5
Therapeutic safety	10 (30.3)
(might)Adverse drug events	10
other	5 (15.1)
Cost-effectiveness of treatment	0
Unnecessary medication	5
Uncertainties requiring further clarification	0
total	33 (100)

**Table 9:** Causes of DRPs.

Classification of causes	Percentage, n (%)
Prescription Issues	20 (60.6)
Inappropriate drug selection	8
Too many types of medication	1
Inappropriate combinations	7
Underdose of drugs	1
Insufficient frequency of administration	3
Drug use problems	13 (39.4)
Patient taking insufficient or no medication	4
Taking medication at the wrong time	2
Patients taking unnecessary medications	3
Patient taking medication incorrectly	4
total	33 (100)

## 4 Discussion

HbA1c levels served as the primary clinical endpoint for this investigation. Regarding the experimental cohort, HbA1c levels decreased from 7.70 ± 2.19% at baseline to 6.50 ± 1.07%, approaching the target level specified in the “Chinese Guidelines for the Prevention and Treatment of Type 2 Diabetes” (2020 Edition), which is ≤6.5% (25, 26). These results are comparable to those observed in two meta-analyses and systematic reviews of pharmacist interventions in diabetes management (21, 27). Additionally, the rate of achieving target HbA1c levels increased from 15.3% at baseline to 45.0%. Patients managed by traditional doctors also exhibited a reduction in HbA1c levels, though the decrease was smaller compared to that observed in the experimental group, with the rate of achieving target levels being only 50% of that seen in the experimental group. Davis (28) noted in a similar study that 46.0% of patients receiving pharmacist collaborative interventions achieved an HbA1c target of<7%, compared to just 23.4% among those who did not receive such interventions. This study’s findings are consistent with those observations. However, a review of related literature indicates that the reductions in glucose levels facilitated by pharmacist collaboration typically range from 1.2% to 2.1%, which is higher than the reductions observed in this study (28–31). The discrepancies might be attributed to variations in the duration of pharmacist interventions across studies, as the effectiveness of glucose reduction tends to correlate positively with the length of intervention. Additionally, inherent differences in study designs (randomized controlled trials versus observational studies utilizing controls) might contribute to varied intervention outcomes. Comparative studies suggest that community pharmacist involvement in diabetes management can yield clinical benefits.

Importantly, the significance of this model lies not only in HbA1c reduction but also in its applicability to the Chinese healthcare system. Given the shortage of physicians and the uneven distribution of resources between tertiary hospitals and community health centers, integrating pharmacists into a hospital–community linkage can provide continuous follow-up, improve adherence, and relieve physician workload. Although the HbA1c reduction was slightly lower than in some international studies, the results highlight that even within China’s resource-constrained primary care settings, pharmacist involvement can substantially enhance diabetes management. Future studies should extend the intervention duration and sample size to validate these findings.

Blood pressure and lipid control indirectly affect glucose levels in patients (32), thus, in addition to assessing glycemic indices, this investigation further examined hypertension and lipid profiles as principal indicators to ascertain the impact of the innovative framework on these health markers. At baseline, both groups had average blood pressure levels within the acceptable range (140/90 mmHg). Following intervention, the treatment group exhibited a statistically meaningful decrease in blood pressure (*P*<0.05), with systolic blood pressure (SBP) decreasing by approximately 10 mmHg and diastolic blood pressure (DBP) by approximately 2mmHg. According to the *2022 standards of the American Diabetes Association* (33), post-management blood pressure in the experimental group reached the target for patients with diabetes and hypertension (<130/80 mmHg) (34). The group not receiving the intervention showed no statistically considerable shifts in blood pressure values. The reduction in the experimental group was similar to findings by Wishah RA et al., where diabetic patients receiving pharmaceutical services showed short-term improvements in SBP and DBP of 4.9-12.1 mmHg and 2.3-7.2 mmHg, respectively (29, 31). Regarding lipid levels, over 12 months, the experimental group showed improvements in total cholesterol (TC), triglycerides (TG), low-density lipoprotein cholesterol (LDL-C), and high-density lipoprotein cholesterol (HDL-C) by 0.76 mmol/L, 0.68 mmol/L, 0.43mmol/L, and 0.15 mmol/L respectively. However, except for LDL-C, no significant between-group differences were found. This suggests that while the pharmacist-led model contributed to modest improvements, its impact on lipid metabolism was limited. Such limitations may be related to the short follow-up period and insufficient lifestyle-focused interventions. Lipid control often requires long-term monitoring, combined dietary and exercise interventions, and possibly more intensive pharmacological adjustments, which were beyond the scope of this model. This indicates that although pharmacological interventions had measurable effects, lifestyle-focused strategies must be strengthened to achieve sustained improvements in lipid control.

From baseline to 12 months, the intervention group demonstrated a significant reduction in BMI of 0.71 kg/m^2^ (P=0.001), whereas no noticeable improvement was observed in the control group. However, both groups remained overweight after the intervention, which suggests that the effect was limited. In another study with a similar 12-month follow-up, a greater reduction in BMI of 1.7 kg/m² (P=0.001) was observed, indicating that more intensive interventions may be required to achieve meaningful weight loss. According to studies by Liu Li (35) and Dai Qiaoyun (36), elevated body mass, encompassing both overweight and obese states, predisposes individuals to T2DM onset and represents a major barrier to effective glycemic control. The China Type 2 Diabetes Prevention and Treatment Guidelines (2020 edition) likewise emphasize that weight reduction in overweight patients is crucial and should be regarded as a core strategy in diabetes management. Therefore, future pharmacist-led interventions could benefit from incorporating more rigorous dietary and exercise components and from involving nutritionists and exercise specialists to deliver multidisciplinary care. Taken together, the BMI and lipid results suggest that while this pharmacist-led model improved glycemic control, its intensity in lifestyle modification was insufficient. From a scalability perspective, structured lifestyle interventions require additional resources, training, and cross-disciplinary collaboration. Without such support, the long-term sustainability and effectiveness of the model may be limited.

The aforementioned indicators suggest that the involvement of pharmacists in the hospital-community diabetes management linkage has a positive effect on the clinical efficacy of patients. Notably, the intervention group began to emphasize the impact of blood pressure and lipids on blood glucose levels, achieving some improvements in the blood pressure and LDL-C levels of diabetic patients, and striving to enhance the comprehensive attainment rates of blood glucose, blood pressure, and lipids. This holistic perspective reflects a shift from focusing solely on glycemic control toward a broader “three-in-one” management approach (glucose, blood pressure, lipids), which is consistent with international guidelines and highlights the pharmacists’ role in comprehensive chronic disease management.

This study established a model involving clinical pharmacists in a tertiary hospital-community diabetes management linkage. Following a year-long treatment regimen involving clinical pharmacists, the 91 T2DM subjects in the experimental cohort demonstrated markedly enhanced achievement rates across various parameters compared to those under conventional physician-led care. The involvement of clinical pharmacists led to increased medication adherence among patients, and the conduct of assessments and analyses of medication-related issues, and DRPs (Drug-Related Problems) status indicated that most of the analyzed DRPs were resolved, suggesting an overall positive effect.

Feasibility and scalability are critical considerations for implementing this model nationwide. While pharmacist training and ongoing supervision represent initial investments, international evidence suggests that pharmacist-led interventions are cost-effective because they reduce complications, hospital admissions, and physician workload (37, 38). In China’s hierarchical healthcare system, pharmacists can play a complementary role to overstretched physicians, particularly in community health service centers. However, widespread implementation requires supportive health policies, sustainable funding, and inclusion of pharmacist services in reimbursement schemes. Cost-effectiveness analyses and health economic evaluations are needed to determine whether the model can be scaled equitably across both urban and rural settings (39).

## 5 Conclusion

In conclusion, this pharmacist-led hospital–community collaborative model significantly enhanced the clinical efficacy and pharmacological management of diabetic patients, while also optimizing the use of medical resources. Its feasibility within China’s tiered healthcare system suggests that it could serve as a scalable reference model for broader implementation. Nevertheless, to maximize its impact, future work should incorporate multidisciplinary teams, extend follow-up durations, and include larger sample sizes to provide stronger evidence for nationwide promotion.

## Data Availability

The original contributions presented in the study are included in the article/**Supplementary Material**. Further inquiries can be directed to the corresponding authors.
