# Supplementary material for: Construction and effectiveness of a pharmacist-involved diabetes management model between tertiary hospitals and community under the hierarchical medical system
Source: Front Clin Diabetes Healthc. 2025 Sep 19;6:1658713. doi: 10.3389/fcdhc.2025.1658713 (PMC12491024; doi:10.3389/fcdhc.2025.1658713)
Supplement: Supplementary file 1 [file DataSheet1.pdf]

补充表 1 糖尿病患者自我行为管理量表

请您根据实际情况在相应的选项“口”内划“√”
（您实际按照要求做的天数）

问题列表	天数							
1.在过去 7 天内，您有几天按糖尿病饮食要求合理安排饮食？	0	1	2	3	4	5	6	7
2.在过去 1 个月内，每周按糖尿病饮食要求合理安排饮食的平均天数？	0	1	2	3	4	5	6	7
3.在过去 7 天内，每天摄入水果/蔬菜达 5 种或 5 种以上的天数？	0	1	2	3	4	5	6	7
4.在过去 7 天内，摄入油腻食物或全脂奶制品的天数？	0	1	2	3	4	5	6	7
5.在过去 7 天内，进行持续时间＞30 分钟的运动情况（包括“散步”）？	0	1	2	3	4	5	6	7
6.在过去 7 天内，进行中等强度活动的情况（包括快走、游泳、骑车等）？	0	1	2	3	4	5	6	7
7.在过去 7 天内，进行血糖监测的天数？	0	1	2	3	4	5	6	7
8.在过去 7 天内，完成适合自身状况的血糖监测次数的天数？	0	1	2	3	4	5	6	7
9.在过去 7 天内，仔细检查自己脚部有无问题的天数？	0	1	2	3	4	5	6	7
10.在过去 7 天内，检查鞋子内部有无异物、平整、舒适度情况的天数？	0	1	2	3	4	5	6	7
11.在过去 7 天内，按医生要求正确服用药物或注射胰岛素的天数？	0	1	2	3	4	5	6	7
12.在过去 7 天内，您是否吸过烟（只吸一口也算在内）？<br>0）否　1）是，如果是，在过去 7 天内，平均一天吸几只烟？——只/天								

补充表 2 密歇根糖尿病知识测试问卷（翻译改良版）

1、糖尿病饮食是：
A 符合大多数中国人的饮食
B 对大多数人来说是健康饮食
C 对多数人来说碳水化合物含量太高
D 对多数人来说蛋白质含量太高

2、以下哪一项碳水化合物含量最高
A 烤鸡
B 瑞士奶酪
C 烤马铃薯
D 花生酱

3、以下哪一项脂肪含量最高？
A 低脂牛奶
B 橘子汁
C 玉米
D 蜂蜜

4、以下哪一项是低糖食品
A 任何无糖食物
B 任何营养食物
C 任何在标签上标明无糖的食物
D 任何每份热量少于 20 卡路里的食物

5、糖化血红蛋白检测反映的是过去多长时间的血糖平均值
A 一天
B 一周
C 12 周
D 6 个月

6、最佳的检测血液葡萄糖浓度的途径是
A 尿检
B 血液检测
C 二者均可

7、无糖果汁对血糖的影响是
A 降低
B 升高
C 不影响

8、下列哪项措施对处理低血糖无效
A 3 块硬糖

B 半杯橘子汁
C 1 杯无糖饮料
D 1 杯脱脂牛奶

9、运动对血糖的影响是
A 降低
B 升高
C 无影响

10、感染会引起
A 血糖升高
B 血糖降低
C 血糖无变化

11、足部护理的最佳方法是
A 每天洗脚并检查足部
B 每天用酒精泡脚
C 每天跑一小时脚
D 买比自己平时大一号的鞋

12、进食低脂食物减少了（）的风险
A 神经病变
B 肾脏病变
C 心脏病变
D 视网膜病变

13、麻木刺痛是（）的特征
A 肾脏病变
B 神经病变
C 视网膜病变
D 肝脏病变

14、以下哪一项和糖尿病没有联系
A 视力问题
B 肾脏问题
C 神经问题
D 肺部问题

15、酮症酸中毒的特征症状包括
A 发抖
B 出汗
C 呕吐
D 低血糖

16、糖尿病人患感冒哪项处理是正确的
A 需要胰岛素量减少
B 少饮水
C 多吃蛋白质类食物
D 加强监测血糖和酮体的频率

17、NPH 胰岛素的作用时间
A 1-3 小时
B 6-12 小时
C 12-15 小时
D 大于 15 小时

18、患者在午餐前发现早餐前胰岛素遗忘注射，应该怎么做
A 不吃午饭降低血糖
B 按照早餐前胰岛素剂量注射
C 按照早餐前剂量两倍进行注射
D 先测血糖，根据血糖决定应该注射多少剂量胰岛素

19、如果患者刚开始有低血糖反应，此时应该
A 锻炼
B 卧床休息
C 喝一些果汁
D 按常规注射胰岛素

20、造成低血糖的原因有
A 胰岛素剂量过大
B 胰岛素剂量过小
C 进食过多
D 运动量过少

21、如果注射了胰岛素却没有进食会引起血糖
A 升高
B 降低
C 保持不变

22、高血糖一般由于
A 胰岛素剂量不足
B 遗忘进食
C 不吃零食
D 尿中有大量酮体

23、下列哪一项最可能引起低血糖
A 大量运动
B 感染

C 饮食过量
D 未注射胰岛素

Supplementary Tables 1 and 2 were translated from their original Chinese versions.

Supplementary Table 1. Summary of Diabetes Self-Care Activities (SDSCA)

Please check "√" in the box corresponding to your actual situation.
(Number of days you actually performed the activity as required)

Question	Days							
1.In the past 7 days, on how many days did you follow a healthy eating plan specific to diabetes?	0	1	2	3	4	5	6	7
2.In the past month, on average, how many days per week did you follow a healthy eating plan specific to diabetes?	0	1	2	3	4	5	6	7
3.In the past 7 days, on how many days did you consume at least 5 servings of fruits/vegetables per day?	0	1	2	3	4	5	6	7
4.In the past 7 days, on how many days did you eat high-fat foods or whole-fat dairy products?	0	1	2	3	4	5	6	7
5.In the past 7 days, on how many days did you perform physical activity lasting more than 30 minutes (including walking)?	0	1	2	3	4	5	6	7
6.In the past 7 days, on how many days did you engage in moderate-intensity activities (such as brisk walking, swimming, cycling)?	0	1	2	3	4	5	6	7
7.In the past 7 days, on how many days did you monitor your blood glucose?	0	1	2	3	4	5	6	7
8.In the past 7 days, on how many days did you perform the recommended number of blood glucose checks appropriate for your condition?	0	1	2	3	4	5	6	7
9.In the past 7 days, on how many days did you carefully examine your feet for problems?	0	1	2	3	4	5	6	7
10.In the past 7 days, on how many days did you check the inside of your shoes for foreign objects, smoothness, and comfort?	0	1	2	3	4	5	6	7
11.In the past 7 days, on how many days did you take your medication or inject insulin exactly as prescribed by your doctor?	0	1	2	3	4	5	6	7
12.In the past 7 days, did you smoke (even one puff counts)? **0) No** **1) Yes**<br>If yes, how many cigarettes did you smoke per day on average? ___ cigarettes/day								

Supplementary Table 2. Michigan Diabetes Knowledge Test (DKT)

1、The diabetes diet is:
A. Suitable for most Chinese people’s diet
B. A healthy diet for most people
C. Too high in carbohydrates for most people
D. Too high in protein for most people

2、Which of the following has the highest carbohydrate content?
A. Roast chicken
B. Swiss cheese
C. Baked potato
D. Peanut butter

3、Which of the following has the highest fat content?
A. Low-fat milk
B. Orange juice
C. Corn
D. Honey

4、Which of the following is a low-sugar food?
A. Any sugar-free food
B. Any nutritious food
C. Any food labeled “sugar-free”
D. Any food with less than 20 calories per serving

5、The HbA1c test reflects the average blood glucose level over what period?
A. One day
B. One week
C. 12 weeks
D. Six months

6、The best way to measure blood glucose concentration is:
A. Urine test
B. Blood test
C. Either one is fine

7、The effect of sugar-free juice on blood glucose is:
A. Lowering
B. Raising
C. No effect

8、Which of the following is NOT effective for treating hypoglycemia?
A. Three pieces of hard candy

B. Half a cup of orange juice
C. One cup of sugar-free drink
D. One cup of skim milk

9、The effect of exercise on blood glucose is:
A. Lowering
B. Raising
C. No effect

10、Infection can cause:
A. Increase in blood glucose
B. Decrease in blood glucose
C. No change in blood glucose

11、The best method of foot care is:
A. Wash and check your feet every day
B. Soak feet in alcohol every day
C. Run for one hour daily
D. Buy shoes one size larger than usual

12、Eating low-fat foods reduces the risk of:
A. Neuropathy
B. Kidney disease
C. Heart disease
D. Retinopathy

13、Numbness and tingling are features of:
A. Kidney disease
B. Neuropathy
C. Retinopathy
D. Liver disease

14、Which of the following is NOT related to diabetes?
A. Vision problems
B. Kidney problems
C. Nerve problems
D. Lung problems

15、The characteristic symptoms of diabetic ketoacidosis include:
A. Shaking
B. Sweating
C. Vomiting
D. Hypoglycemia

16、When a person with diabetes catches a cold, the correct management is:
A. Reduce insulin dose
B. Drink less water
C. Eat more protein foods
D. Increase the frequency of blood glucose and ketone monitoring

17、The duration of action of NPH insulin is:
A. 1–3 hours
B. 6–12 hours
C. 12–15 hours
D. More than 15 hours

18、If a patient realizes before lunch that they forgot to inject insulin before breakfast, what should they do?
A. Skip lunch to lower blood glucose
B. Inject the usual breakfast insulin dose
C. Inject twice the usual breakfast insulin dose
D. Test blood glucose first, then decide on the insulin dose

19、If a patient just begins to experience hypoglycemia, what should they do?
A. Exercise
B. Rest in bed
C. Drink some fruit juice
D. Inject insulin as usual

20、Causes of hypoglycemia include:
A. Insulin overdose
B. Too little insulin
C. Overeating
D. Too little exercise

21、If insulin is injected without eating, blood glucose will:
A. Increase
B. Decrease
C. Remain unchanged

22、Hyperglycemia is generally caused by:
A. Insufficient insulin dosage
B. Forgetting to eat
C. Not eating snacks
D. Large amounts of ketones in urine

23、Which of the following is most likely to cause hypoglycemia?
A. Vigorous exercise

B. Infection
C. Overeating
D. Not injecting insulin

Supplementary Table 3.

Specialized training received by community pharmacists

Training module	Delivery mode	Frequency
Establishment of chronic-disease records	Online and in-person lectures	3
Prescription review	Face-to-face training	1
Pharmacotherapy consultation & patient education	Pharmacy ward-round practicum	2
Home medicine cabinet management	In-person lecture	1
Adverse drug reaction monitoring	In-person lecture	1
Knowledge of diabetes complications	Online lecture	1
Advances in diabetes & novel pharmacotherapy	Online guideline-based session	1
Use of insulin (including pumps)	Face-to-face training	2
Lifestyle education	Online lecture	3
Blood glucose monitoring	Online lecture	3
